# Tuberculosis and Poverty: Why Are the Poor at Greater Risk in India?

**DOI:** 10.1371/journal.pone.0047533

**Published:** 2012-11-19

**Authors:** Olivia Oxlade, Megan Murray

**Affiliations:** 1 Department of Epidemiology, Harvard School of Public Health, Boston, Massachusetts, United States of America; 2 Division of Global Health Equity, Brigham and Women's Hospital, Boston, Massachusetts, United States of America; 3 Infectious Disease Unit, Massachusetts General Hospital, Boston, Massachusetts, United States of America; Institut de Pharmacologie et de Biologie Structurale, France

## Abstract

**Background:**

Although poverty is widely recognized as an important risk factor for tuberculosis (TB) disease, the specific proximal risk factors that mediate this association are less clear. The objective of our study was to investigate the mechanisms by which poverty increases the risk of TB.

**Methods:**

Using individual level data from 198,754 people from the 2006 Demographic Health Survey (DHS) for India, we assessed self-reported TB status, TB determinants and household socioeconomic status. We used these data to calculate the population attributable fractions (PAF) for each key TB risk factor based on the prevalence of determinants and estimates of the effect of these risk factors derived from published sources. We conducted a mediation analysis using principal components analysis (PCA) and regression to demonstrate how the association between poverty and TB prevalence is mediated.

**Results:**

The prevalence of self-reported TB in the 2006 DHS for India was 545 per 100,000 and ranged from 201 in the highest quintile to 1100 in the lowest quintile. Among those in the poorest population, the PAFs for low body mass index (BMI) and indoor air pollution were 34.2% and 28.5% respectively. The PCA analysis also showed that low BMI had the strongest mediating effect on the association between poverty and prevalent TB (12%, p = 0.019).

**Conclusion:**

TB control strategies should be targeted to the poorest populations that are most at risk, and should address the most important determinants of disease—specifically low BMI and indoor air pollution.

## Introduction

With 9 million incident cases and 2 millions deaths reported in 2009, Tuberculosis (TB) continues to cause significant morbidity and mortality, especially in the low and middle income countries (LMIC) identified by the World Health Organization (WHO) as the 22 “high burden” nations [1]. While TB incidence has declined in most regions of the world, the slow pace of progress has prompted a search for new targets for interventions [2]. Recent work suggests that, on a national level, TB trends track more closely with social and economic indicators than with measures of TB control activities [3], [4]. These data imply that the targeting of interventions to the most vulnerable groups may be necessary to speed progress toward elimination of this scourge.

There is substantial evidence that poverty is a determinant of TB, both at the macro-scale and in individual and hierarchical analyses. Janssens and Rieder documented a linear association between per capita GDP [5] and TB incidence, and Dye found that the country level human development index was a strong predictor of changes in TB incidence over time [4]. Although several studies report discrepant findings [6]–[8], most analyses of data have confirmed the positive association between household and area poverty indicators and TB in such diverse settings as South Africa [9], Brazil [10], Vietnam [11] and Zambia [12]. Among the social, environmental and biological determinants of TB, many are more prevalent among the poor than in wealthier groups and these determinants likely contribute to a complex web of poverty-based risk factors that is difficult to tease apart.

With the recognition of poverty as a root cause of TB [13]–[16], the need to intervene not only on economic status, but also on the proximal risk factors that put the poor at risk is increasingly clear. Several groups have described frameworks that suggest how and when common proximate risk factors act on the TB pathogenetic pathway that includes exposure, infection, active disease and eventual disease outcomes [14], [17]. Although some epidemiologic studies have sought to measure the impacts of these determinants, only a few have addressed this question in the context of understanding the routes by which poverty leads to TB [12]. The objective of our study was to investigate the mechanisms by which poverty increases the risk of TB, using data from a large population based survey in India.

## Methods

### Ethics Statement

Ethics exemption waiver issued by Harvard School of Public Health as Demographic Health Survey (DHS) data set is a public data set.

### Prevalence of TB and risk factors for TB in Indian DHS

Individual level data on TB status at the time of interview and risk factors were obtained from the DHS for India conducted in 2005–2006. This publically available cross-sectional survey (described at http://www.measuredhs.com/) assessed a range of risk factors in a weighted sample of 198,754 individuals aged 15–49.

### Description and Classification of Key Variables

We assessed the prevalence of self-reported TB, i.e. those who responded to the question, “do you suffer from tuberculosis?” affirmatively. We also assessed the following previously identified common and modifiable TB risk factors [16]: Smoking and tobacco use, indoor air pollution (IAP), low BMI (<18.5 kg/m^2^), Diabetes Mellitus (DM), alcohol use and HIV. Other social and demographic variables assessed included age, gender, household density, access to health insurance and rural versus urban dwelling. Where possible, we chose exposure categories to be consistent with those for which we had quantitative summaries of effects on TB risk. We classified smoking as any current cigarette smoking. We considered participants to be exposed to IAP if they used bio-mass cooking fuel used in the home [18]. We classified low BMI according to internationally recognized cut offs where low BMI is defined as <18.5 kg/m^2^ and normal BMI as 18.5–24.9 kg/m^2^ [19]. We classified alcohol use based on the frequency of use (daily or otherwise). We classified DM by self-report.

### Measuring Socio Economic Status

We used the “DHS Wealth Index factor” score as an indicator of household socio economic status (SES). This index assesses household's ownership of such assets as a television and a car, materials used in housing, drinking water source, and toilet facilities. Assets are assigned weights based on principal components analysis and the resulting scores are standardized to a standard normal distribution. Households are then scored and divided into population quintiles. Asset based measures allow ranking of households without consumption data and have been shown to be more stable, i.e. less sensitive to transient fluctuation, than consumption data [20], [21].

### Statistical analysis

All statistical analyses were conducted using SAS for Windows, Version 9.2 and were adjusted for the DHS sampling strategy using survey weights.

#### TB prevalence

We estimated the prevalence of self reported TB stratified by SES quintile.

#### Effect estimates of TB determinants

To determine the population attributable fraction (PAF) of TB due to key risk factors in different SES strata, we used published estimates of effect. Estimates were derived from meta-analyses for smoking, IAP, DM and alcohol use [22]–[24]. The summary estimate of effect for alcohol use/abuse corresponds to “>40 g of alcohol per day” and “alcohol use disorder” [24], in contrast to the exposure reported in the DHS data: daily alcohol intake. Because there is no meta-analysis available on the effect of HIV on TB risk, we used an estimate published by the WHO [25]. Similarly, because our exposure category for low BMI differed from the very low BMI exposure for which a summary effect estimate is available [26], we used instead an estimate from one of the studies included in the systematic review [27]. To confirm that these factors also play a role in India, we performed a sensitivity analysis comparing the adjusted effect estimates from DHS data with published estimates and determined whether the published estimates fell within the 95% confidence intervals of the estimates from DHS.

#### Prevalence of TB risk factor by SES and Calculation of the PAF

We estimated the prevalence of the proximate risk factors by three SES strata: the poorest 40%, the middle 40%, and the richest 20% [21]. We then used the prevalence of each TB risk factor and the published estimates of effect to calculate a PAF for each SES strata, based on the formula: PAF = [prevalence of risk factor* (RR-1)]/[prevalence *(RR-1)+1].

#### Principal component analysis and Mediation analysis

To identify the factors that mediate the relationship between poverty and TB, we conducted a mediation analysis. Recognizing that many of the variables associated with TB risk may be tightly correlated and that this collinearity can reduce the power of multiple regression models, we used principal components analysis (PCA) to group correlated variables together into a smaller group of uncorrelated components. Components were retained if they displayed Eigen values of greater than one. A threshold of 0.39 was used to determine if and where components loaded. The uncorrelated components were then assessed for evidence of mediation using a series of multivariate regression models, where each individual principal component was added to a minimal model. This approach is based on the principal that controlling for intermediates in the causal pathway between a risk factor and an outcome will reduce the observed effect of the more distal determinant [28]. Evidence of mediation was formally assessed using the Relative Mediation Effect (RME) [28], [29] which represents the proportion of the association between poverty and TB prevalence that was accounted for by each potentially mediating component [29]. Statistical testing and construction of percentile confidence intervals around each RME was done by bootstrapping 1000 iterations [30]. Of note, we did not include HIV in this analysis since this variable was available for only a fraction of the cohort.

## Results

**Figure 1:**
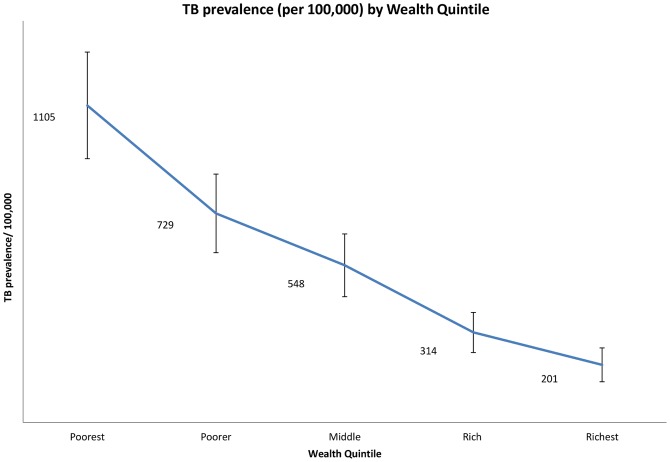
Self Reported TB prevalence (per 100,000) by wealth quintile.

**Figure 2:**
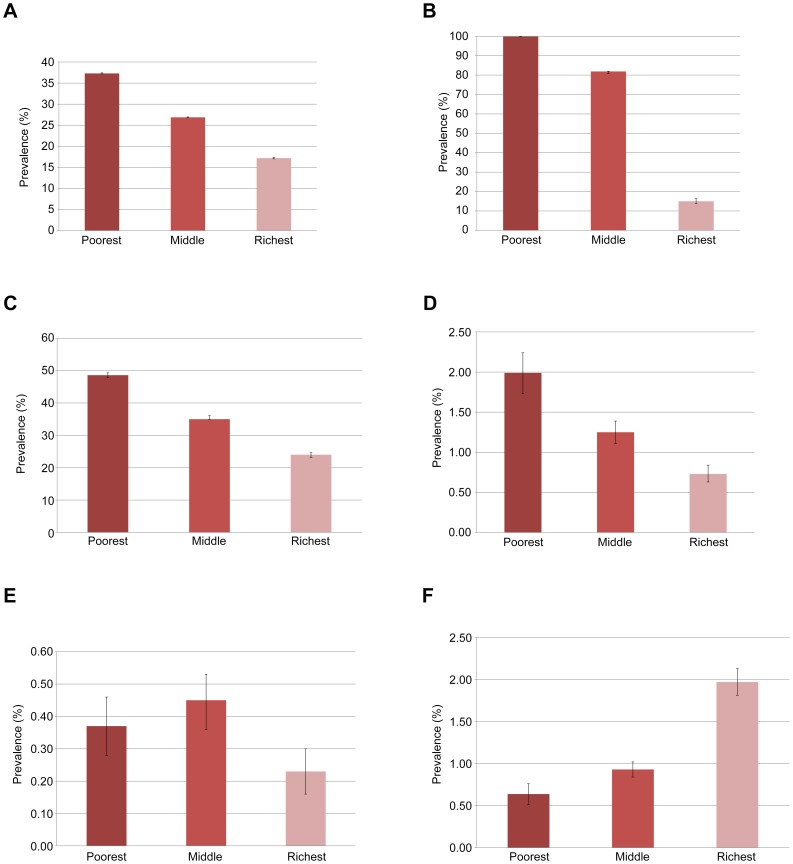
Prevalence of Key TB Risk factors (and 95% Confidence Interval) by wealth level*. Figure 2a. Tobacco Use (Cigarettes, chewing tobacco or other). Figure 2b. Exposure to Indoor Air Pollution. Figure 2c. Low BMI (less than 18.5 kg/m^2^). Figure 2d. Alcohol Use (daily). Figure 2e. HIV seroprevalence. Figure 2f. Diabetes Mellitus. FOOTNOTE TO FIGURES 2a–f: * Wealth level or socio economic status is divided as follows: poorest 40%, middle 40%, and richest 20%.

The prevalence of self reported TB in India was 545 per 100,000 (95% CI 493–597 per 100,000). Figure 1 shows that TB prevalence increases linearly with wealth quintile, with estimates ranging from 201 per 100,000 population (95% CI 142–260 per 100,000) among the wealthiest to 1105 per 100,000 population (95% CI 919–1291 per 100,000) in the poorest. Figure 2A–F shows that most risk factors for TB are more prevalent among the poor than in the wealthier strata, with the exceptions of DM, which is highest in the wealthiest population, and HIV, which is evenly distributed among the low and middle strata but is less common in the wealthiest quintile.

Table 1 compares the summary effect estimates reported from published sources with the adjusted odds ratios for proximal determinants of TB in the DHS data set. Published effect estimates fall within the 95% confidence intervals of the adjusted estimates for DHS for all variables except cigarette and alcohol use and BMI. As above, alcohol use/abuse was differently classified in the DHS than in the meta-analyses. Although the impact of HIV on TB prevalence was less than reported estimates of its impact on incidence, previous studies have noted the marked discrepancy between the relative risk of HIV for TB incidence and prevalence [12], and our estimates were consistent with other reports on prevalence [12], [31]–[33], which have not, to date, been summarized in a systematic review or meta-analysis.

**Table 1:** Association between proximate risk factors and Tuberculosis.

Risk Factor	Univariate OR and 95% CI (DHS)	Multivariate Odds Ratio (aOR) and 95% CI (DHS)	Relative Risk and 95% Cl for Key Risk factors (from published source)	Reference
Smoking Cigarettes	1.49 (1.16–1.90)	0.77 (0.56–1.06)	2.0 (1.6–2.5)	[22]
Chewing Tobacco	2.12 (1.66–2.72)	1.38 (1.03–1.86)	-	
Indoor Air Pollution (IAP)	3.07 (2.36–4.01)	2.00 (1.35–2.98)	1.4 (0.6–3.4)	[22]
Low Body Mass Index (BMI)	2.90 (2.39–3.51)	3.71 (2.84–4.83)	2.06 (1.46–2.91)	[27]
Alcohol Use- Daily	1.98 (1.16–3.37)	1.36 (0.73–2.55)	2.9 (1.9–4.6)	[24]
HIV Sero-prevalence	5.75 (2.46–13.43)	4.72(2.0–11.20)	26.7 (20–35)	[25]
Diabetes Mellitus	2.77 (1.67–4.59)	4.89 (2.73–8.76)	3.1 (2.3–4.3)	[23]
Age (per year)	1.04 (1.03–1.05)	1.06 (1.04–1.07)	-	
Male Gender	1.72 (1.43–2.05)	1.83 (1.37–2.4)	-	
Household density (rooms for sleeping/people sleeping)	1.11 (1.07–1.15)	1.08 (1.03–1.14)	-	
Family member with health insurance	0.46 (0.27–0.77)	0.59 (0.29–1.23)	-	
Rural dwelling	1.86 (1.50–2.30)	0.91 (0.68–1.21)	-	

Full multivariate regression model includes all risk factors shown above as well additional variables “smoking other than cigarettes/tobacco”, “frequent meat intake” and “frequent fresh produce intake”.

Low BMI was calculated to have the highest PAF at 34.2% in the lowest wealth category, followed by IAP with a PAF of 28.5%, and cigarette smoking with a PAF of 16.0% (Table 2). Low BMI also had the largest PAF at 20.3% among the wealthiest category. Diabetes was the only risk factor that was predicted to have a greater impact in the wealthier strata, with a PAF of 4.0%.

**Table 2:** Population attributable risk fractions by wealth category- Using published relative risk estimates.

Risk factors	Relative Risk*	Prevalence of risk factor			Population Attributable Fractions		
		Poorest	Middle	Richest	Poorest	Middle	Richest
Smoking cigarettes	2.0	19.0%	11.0%	6.0%	16.0%	9.9%	5.7%
Indoor Air Pollution	1.4	99.9%	82.0%	15.0%	28.5%	24.7%	5.7%
Low BMI	2.1	49.0%	35.0%	24.0%	34.2%	27.1%	20.3%
Alcohol use (daily)	2.9	2.0%	1.0%	0.7%	3.7%	1.9%	1.3%
Diabetes Mellitus**	3.1	0.6%	1.0%	2.0%	1.3%	2.1%	4.0%
HIV Seroprevalence	26.7	0.4%	0.5%	0.2%	8.7%	10.4%	5.6%

* Reference for published estimate of each risk factor provided in Table 1.

** Estimates from other sources suggests that self reported prevalence of diabetes is underreported in population (see text for more detail).

PCA transformed 20 out of 21 variables into the following six uncorrelated components (Table 3); 1) protein intake; 2) educational achievement; 3) tobacco use, alcohol use and male gender; 4) rural setting, exposure to indoor air pollution and health insurance; 5) intake of fresh produce, and 6) BMI, anemia, milk intake and DM. (For this component, anemia was negatively but strongly correlated with BMI, milk intake and DM and that this component therefore likely reflects dietary fat/protein intake). A full list of variables initially included in the PCA, the rotated factor pattern, and the percent of explained variance accounted for by the retained components are included in Table S1A–C in Appendix S1.

**Table 3:** Uncorrelated components derived from Principal Components Analysis (PCA).

Component number	Component name	Variables (and direction) included in component
1	Protein intake	fish (eat often) meat (eat often) and eggs (eat often)
2	Educational achievement	Education (more educated) literacy (more literate)
3	Tobacco use, alcohol use and male gender	Alcohol (drinks more often) Smokes cigarettes (yes) smokes tobacco (yes) smokes other (yes) gender (male)
4	Rural setting & exposure to IAP	setting (rural) exposure to indoor air pollution (yes) [someone in family has health insurance (more likely)] (−ve)
5	Fresh produce intake	beans (eat often) green vegetable (eat often)
6	BMI, anemia, milk intake and DM	BMI (underweight) [Diabetes (yes)] (−ve) [milk (eat often)]; (−ve) anemia (more likely)

Table 4 shows that principal component 6 (BMI, anemia, milk intake and DM) has the greatest relative mediation effect (RME = 11.9%, p = 0.019) on the impact of poverty on TB prevalence. Although Component 2 (educational achievement) also had an RME value of 6.6%, this effect was not statistically significant.

**Table 4:** Mediation effects of Principal Components for the association between Tuberculosis and Socio Economic Status(SES).

MODEL	Adjusted OR for SES	Relative Mediation Effect (RME) (%)*	P value
Baseline model (age and SES)	5.43		
Baseline plus Protein intake	5.38	0.55%	0.85
Baseline plus Educational achievement	4.86	6.55%	0.43
Baseline plus Tobacco use, alcohol use and male gender	5.18	2.79%	0.34
Baseline plus Rural setting & exposure to IAP	5.54	−1.19%	0.92
Baseline plus Fresh produce intake	5.06	4.17%	0.24
Baseline plus BMI, anemia, milk intake and DM	4.44	11.9%	0.019

* Each model is compared to the Baseline model that includes age and SES.

## Discussion

DHS data shows that members of the poorest quintile in India are at a 5.5-fold higher risk for self- reported prevalent TB than those in the wealthiest quintile. The TB prevalence rate of 1105 per 100,000 in this group exceeds those estimated in high HIV burden populations like South Africa where the national prevalence of TB was reported to be 782/100,000 in 2009 [1] and the prevalence of HIV is 18% [34]. In contrast, the HIV prevalence among the poorest quintile in India was only 0.4%. Almost all known TB risk factors were more common among the Indian poor, with the notable exceptions of DM and HIV. Those in the poorest strata frequently share multiple risk factors for active TB, substantially increasing their risk. Among the 49% of the poorest quintile who share the two risk factors, low BMI and indoor air pollution, TB risk is increased almost 5 fold compared to the less than 1% in the same strata without either risk factor (results not shown). Low BMI was found to have be the strongest mediator of the association between TB and poverty, and was responsible for the largest PAF for TB in all income levels with biomass fuel exposure a close second in all but the wealthiest sector of the population.

The finding that the poor have many overlapping risk factors for TB is supported by various theoretical models described in the literature [15], [16]. Changes to structural determinants of TB epidemiology at a global level (including population mobility, rapid urbanization and population growth) have given rise to an unequal distribution of many of the social determinants considered in this study [15]. People with low SES typically live in poor housing and environmental conditions, have greater food insecurity and have less access to quality health care relative to those from higher SES groups [16]. All of these social determinants are also related to TB, and often work together to put the poor at greater risk of disease by acting on different stages in the pathogenetic pathway.

Although the strong association between per capita GDP and TB incidence has been well established both on a global and regional level [3], [4] and both ecological and hierarchical analyses within countries have identified low SES as a strong risk factor for TB [35], [36], few studies have attempted to quantitatively assess how poverty creates this risk. In one recent study, Boccia et al. used mediation analysis to identify the proximal causes that resulted in more than a six fold increase in TB risk in the poorest sectors of a Zambian population [6]. These investigators then evaluated the impact of blocks of risk factors for TB from their conceptual framework and found that reduced food intake variables were the only ones that emerged as mechanisms mediating the causal relationship between poverty and TB. Like Boccia and others, we recognized that the risk factors that may mediate the association between poverty and TB are often jointly distributed. We also addressed this issue by grouping these risk factors into clusters or blocks but did so agnostically, by performing a PCA to identify determinants that co-vary. The resulting clusters included several discrete types of risk factors such educational achievement, tobacco and alcohol, and exposure to biomass cooking fuel. Interestingly, however, nutritional variables were grouped into three different uncorrelated clusters, two of which reflected intake of protein and fresh produce respectively and a third which reflected a strong positive correlation between underweight and anemia, and a strong negative correlation between these two markers of poor general nutritional status and diabetes and milk intake. These data suggest that the nutritional determinants of TB are complex and multifactorial.

Another study by Boccia et al. considered PAFs for risk factors, but included only those determinants and effect estimates that were identified as risk factors in the study data, which was limited by a very small sample size [12]. In contrast to this approach, we relied on previously reported effect estimates and estimated PAFs for different socio-economic sectors based on the prevalence of these risk factors in the India DHS data. We assumed that the observed association would also exist in India and confirmed that most of these summary effects were consistent with those in our data set. Nonetheless, we recognized that some risk factors for incident TB might reduce survival and thereby decrease disease prevalence. This is particularly true for HIV which is a strong risk factor not only for TB but also for death. Previous studies have shown that HIV increases the TB prevalence 2–3 fold in contrast to its 20 fold impact on TB incidence [25]. Some behavioral risk factors such as smoking and alcohol use might be altered by TB disease and would be better assessed in cohort studies in which these exposures were assessed prior to onset of TB [37]. Lastly, we recognized that since TB was classified on the basis of self-report, this might lead to bias if wealthier individuals are more likely to know or report their TB status. Because of these methodological issues, we used published estimates even when these deviated from the estimated association in the DHS data.

Our study has several limitations. First, DHS data relies on self-report for most variables with the exception of HIV status, anemia and anthropomorphic measures. Most notably, DM is self-reported and the prevalence of 3.5% reported in our study was much lower than the 11% recently reported by Danaei et al [38] who used measures of hyperglycemia to confirm the diagnosis. When we used this higher prevalence estimate for DM to estimate the PAF for India, we found that it increased to almost 20%. However, since DM prevalence was not stratified by SES status in the Danaei study, it is not possible to estimate the PAF for rich and poor communities respectively. TB status was also determined by self-report and we found that the prevalence of self reported TB (545 per 100,000) is substantially higher than the estimated prevalence of TB reported by WHO in 2006 (299 per 100,000) [39]. However, no recent prevalence survey has been conducted in India and in the absence of actual prevalence assessments, WHO prevalence estimates are derived from case notifications, with correction factors for the expected case detection rate and the mean duration of disease. Thus, the discrepancy between these estimates may reflect the fact that a large portion of TB patients who are diagnosed and treated in the private sector in India and who are thus not notified as cases. Importantly, one of the last prevalence surveys done nationally in 2000 estimated a prevalence of 846 per 100,000 in marked contrast to the 458 per 100,000 estimate reported by the WHO in this same period [40].

Secondly, although the data are cross-sectional, we assumed that SES is a determinant of prevalent TB rather than that prevalent TB affects economic status. Although there is clear evidence that out-of-pocket TB treatment expenses can result in catastrophic spending in India and elsewhere [41]–[44], we chose an asset based socio-economic assessment that we believe is less likely to vary in relation to prevalent health shocks than consumption based measures.

Here, we reported PAFs in order to provide an estimate of how much disease in an exposed population might be prevented by removing a specific exposure. This measure is meant to capture the fraction of the disease burden that could be prevented by eliminating a specific risk factor. In our analysis, the sum of the individual PAFs were found to approach 100% in the poorest stratum while the sum of individual PAFs was less than 50% in the rich strata. These findings suggest that it will be necessary to target multiple proximal risk factors to achieve a lasting impact among this vulnerable population.

Our finding that the prevalence of TB was substantially higher in the poorest stratum in India is one that can likely be replicated in any of the 22 high TB burden countries. That the poor are also the sector that are least likely to access TB diagnosis and care emphasizes the need to improve and focus TB services on this group. While the alleviation of poverty globally may be slow to be realized in the current economy, specific measures designed to reduce TB among the poor can be implemented. For example, new but more costly diagnostics that enable earlier detection and treatment may be unlikely to reach poor populations without specific policies that target these interventions to those most in need. The targeting of active case finding to the poor who share multiple risk factors may be a more successful strategy than the focusing on those with individual risk factors. On a more global scale, the existing TB control targets set by the World Health Assembly in 1991, to detect 70% of TB cases and successfully treat 85% of those detected, allow for neglect of the most vulnerable populations; revision of these goals to explicitly address equity could enable the development and financing of TB control strategies aimed at the poor. In addition to diagnosis and care, TB prevention should be tailored to address the most important determinants contributing to TB among the poor - in this setting, specifically low BMI and indoor air pollution. Given the complexity of the interactions between multiple risk factors, it will also be essential to consider the root causes of these proximal determinants. Our findings support the approach recently endorsed by the Stop TB Department at the WHO [13], [16] that a comprehensive and integrated approach to disease control that simultaneously targets specific risk factors and poverty reduction more widely should be supported and prioritized in order to reduce TB mortality and morbidity.

## Supporting Information

Appendix S1 **Supplemental Results.** Table S1a. Principal components Analysis. Rotated Factor Pattern for all variables initially included in the PCA, using Varimax Rotation Method. Table S1b. Principal Components Analysis. Percent of variance explained by 6 Principal Components. Table S1c. Principal Components Analysis. Final Communality Estimates (Total = 11.188779).
(DOCX)
