# Supplementary material for: Tuberculosis and Poverty: Why Are the Poor at Greater Risk in India?
Source: PLoS One. 2012 Nov 19;7(11):e47533. doi: 10.1371/journal.pone.0047533 (PMC3501509; doi:10.1371/journal.pone.0047533)
Supplement: Appendix S1 — Supplemental Results. Table S1a. Principal components Analysis. Rotated Factor Pattern for all variables initially included in the PCA, using Varimax Rotation Method. Table S1b. Principal Components Analysis. Percent of variance explained by 6 Principal Components. Table S1c. Principal Components Analysis. Final Communality Estimates (Total = 11.188779). (DOCX) [file pone.0047533.s001.docx]

**SUPPORTING INFORMATION LEGENDS:**

**Supplemental Results Table S1a. Principal components Analysis.**  Rotated Factor Pattern for all variables initially included in the PCA, using Varimax Rotation Method.

**Supplemental Results Table S1b. Principal Components Analysis.** Percent of variance explained by 6 Principal Components.

**Supplemental Results Table S1c. Principal Components Analysis.** Final Communality Estimates (Total = 11.188779).

Table S1a. Principal components Analysis- Rotated Factor Pattern, using Varimax Rotation Method

| **Rotated Factor Pattern** | | | | | | | | | | | | |
| --- | --- | --- | --- | --- | --- | --- | --- | --- | --- | --- | --- | --- |
|  | **Component**  **1** |  | **Component**  **2** |  | **Component 3** |  | **Component 4** |  | **Component 5** |  | **Component 6** |  |
| **GENDER** | 3 |  | 31 |  | 75 | * | 9 |  | -1 |  | -15 |  |
| **LOW BMI** | -2 |  | 1 |  | 5 |  | 24 |  | 0 |  | 58 | * |
| **CROWDING** | -6 |  | -32 |  | 6 |  | 15 |  | 6 |  | 6 |  |
| **RURAL SETTING** | -8 |  | -18 |  | 0 |  | 76 | * | -6 |  | 9 |  |
| **DIABETES** | 4 |  | -10 |  | -1 |  | -10 |  | -7 |  | -39 | * |
| **FREQUENCY of ALCOHOL use** | 16 |  | -8 |  | 68 | * | -3 |  | -4 |  | -12 |  |
| **SMOKE_Cigg** | 3 |  | -2 |  | 67 | * | 10 |  | -4 |  | -22 |  |
| **SMOKE_Tobbaco** | 1 |  | -11 |  | 54 | * | -3 |  | -2 |  | 23 |  |
| **SMOKE_Other** | -1 |  | -4 |  | 51 | * | -5 |  | 6 |  | 18 |  |
| **FREQUENCY of MILK intake** | -14 |  | 33 |  | -4 |  | 3 |  | 39 | * | -43 | * |
| **FREQUENCY of BEANS intake** | -5 |  | 4 |  | 2 |  | -7 |  | 76 | * | 2 |  |
| **FREQUENCY of GREEN VEG intake** | 8 |  | -4 |  | -2 |  | -6 |  | 74 | * | 10 |  |
| **FREQUENCY of FRUIT intake** | 14 |  | 37 |  | -8 |  | -30 |  | 35 |  | -26 |  |
| **FREQUENCY of EGGS intake** | 87 | * | 7 |  | 8 |  | -1 |  | 6 |  | -3 |  |
| **FREQUENCY of FISH intake** | 87 | * | 3 |  | 4 |  | 2 |  | -6 |  | 4 |  |
| **FREQUENCY of MEAT intake** | 89 | * | 0 |  | 7 |  | -2 |  | 3 |  | -3 |  |
| **LITERACY LEVEL** | 2 |  | 91 | * | 3 |  | -13 |  | 5 |  | 6 |  |
| **EDUCATION ATTAINMENT** | 0 |  | 91 | * | 0 |  | -22 |  | 8 |  | 1 |  |
| **INDOOR AIR POLLUTION** | 2 |  | -34 |  | 7 |  | 73 | * | -11 |  | 15 |  |
| **Access to family HEALTH INSURANCE** | -5 |  | 6 |  | 3 |  | -55 | * | 3 |  | -3 |  |
| **ANEMIA** | 3 |  | -26 |  | -17 |  | -13 |  | 0 |  | 51 | * |
| **Printed values are multiplied by 100 and rounded to the nearest integer. Values greater than** \|**0.39**\| **are flagged by an '*'.** | | | | | | | | | | | | |

Table S1b. Principal Components Analysis. Variance explained by each Component.

| **Component** | **Variance Explained by each component** |
| --- | --- |
| **1** | 2.3801392 |
| **2** | 2.3503737 |
| **3** | 2.0915364 |
| **4** | 1.6978730 |
| **5** | 1.4576837 |
| **6** | 1.2111730 |

Table S1c. Principal Components Analysis. Final Communality Estimates (Total = 11.188779).

|  | **Final Communality Estimates** |
| --- | --- |
| **GENDER** | 0.68298884 |
| **LOW BMI** | 0.39224854 |
| **CROWDING** | 0.14241175 |
| **RURAL SETTING** | 0.62344931 |
| **DIABETES** | 0.17492306 |
| **FREQUENCY of ALCOHOL use** | 0.51394779 |
| **SMOKE_Cigg** | 0.51602728 |
| **SMOKE_Tobbaco** | 0.36015707 |
| **SMOKE_Other** | 0.30037547 |
| **FREQUENCY of MILK intake** | 0.46717166 |
| **FREQUENCY of BEANS intake** | 0.58800407 |
| **FREQUENCY of GREEN VEG intake** | 0.57278206 |
| **FREQUENCY of FRUIT intake** | 0.44385875 |
| **FREQUENCY of EGGS intake** | 0.76535015 |
| **FREQUENCY of FISH intake** | 0.75746052 |
| **FREQUENCY of MEAT intake** | 0.79645305 |
| **LITERACY LEVEL** | 0.84831864 |
| **EDUCATION ATTAINMENT** | 0.87650383 |
| **INDOOR AIR POLLUTION** | 0.68361203 |
| **Access to family HEALTH INSURANCE** | 0.30773273 |
| **ANEMIA** | 0.37500236 |
